# Anchoring of both PKA-RIIα and 14-3-3θ regulates retinoic acid induced 16 mediated phosphorylation of heat shock protein 70

**DOI:** 10.18632/oncotarget.3702

**Published:** 2015-03-30

**Authors:** Cui-Ling Ding, Gang Xu, Hai-Lin Tang, Shi-Ying Zhu, Lan-Juan Zhao, Hao Ren, Ping Zhao, Zhong-Tian Qi, Wen Wang

**Affiliations:** ^1^ Department of Microbiology, Shanghai Key Laboratory of Medical Biodefense, Second Military Medical University, Shanghai, China

**Keywords:** RAI16, Fam160B2, AKAP, PKA-RIIα, 14-3-3θ

## Abstract

Our previous study reported that retinoic acid induced 16 (RAI16) could enhance tumorigenesis in hepatocellular carcinoma (HCC). However, the cellular functions of RAI16 are still unclear. In this study, by immunoprecipitation and tandem (MS/MS) mass spectrometry analysis, we identified that RAI16 interacted with the type II regulatory subunit of PKA (PKA-RIIα), acting as a novel protein kinase A anchoring protein (AKAP). In addition, RAI16 also interacted with heat shock protein 70 (HSP70) and 14-3-3θ. Further studies indicated that RAI16 mediated PKA phosphorylation of HSP70 at serine 486, resulting in anti-apoptosis events. RAI16 was also phosphorylated by the anchored PKA at serine 325, which promoted the recruitment of 14-3-3θ, which, in turn, inhibited RAI16 mediated PKA phosphorylation of HSP70. These findings offer mechanism insight into RAI16 mediated anti-apoptosis signaling in HCC.

## INTRODUCTION

PKA signaling is important for the post-translational modification of proteins [1, 2]. PKA activity is spatially and temporally regulated through compartmentalization by protein kinase A anchoring proteins (AKAPs) [3]. One important function of AKAP is to assemble signaling complexes containing multiple kinases, phosphatases and regulatory proteins [4]. By simultaneously interacting with multiple signaling enzymes, AKAPs can integrate diverse transduction pathways that coordinately regulate the function of specific cellular substrates [5]. Due to the importance of phosphorylation in cellular functions, it is perhaps not surprising that distinct AKAPs coordinate aspects of mitosis and cancer progression [6, 7]. For example, AKAP-4 was also highly expressed in prostate cancer cells and served as a potential target for prostate cancer adoptive immunotherapy or anti-tumor vaccination [8, 9]. AKAP10 was significantly increased in colorectal cancer patients and polymorphism (2073 A/G, I646V) was associated with colorectal cancer risk [10]. AKAP-3 appeared to be tumor-restricted expression and associated with worse overall survival of epithelial ovarian cancer [11]. AKAP12 inhibited oncogenic proliferation, invasion, chemotaxis and neovascularization [12]. Down regulation of AKAP12 that functioned as a tumor suppressor gene had been found in association with lung adenocarcinoma, breast carcinoma, gastric carcinoma, esophageal carcinoma, and acute leukemia [13-15].

Our previous study reported that retinoic acid induced 16 (RAI16) could enhance tumorigenesis in hepatocellular carcinoma (HCC) as the target of miR-483-5p. The cells over-expressing RAI16 showed resistant to apoptosis to some extent [16]. However, the mechanism of anti-apoptosis of RAI16 is still unclear. In the present study, we identified RAI16 as a novel AKAP. We found that RAI16 mediated PKA phosphorylation of HSP70 and the phosphorylation of RAI16 by the anchored PKA promotes the recruitment of 14-3-3θ, which, in turn, inhibits RAI16 mediated PKA phosphorylation of HSP70. These findings offer mechanism insight into RAI16 mediated anti-apoptosis signaling.

## RESULTS

### RAI16 interacts directly with PKA-RIIα

We performed a proteomic screen for interaction partners of RAI16. Lysates from HEK293T cells expressing Flag-tagged RAI16 were incubated with anti-Flag antibodies, immunoprecipitated complexes were isolated and associated proteins were identified by tandem (MS/MS) mass spectrometry. Detection of peptides from RAI16 was used as quality controls. Peptides from PKA-RIIα, HSP70 and 14-3-3θ were also identified (Figure S1).

**Figure 1:**
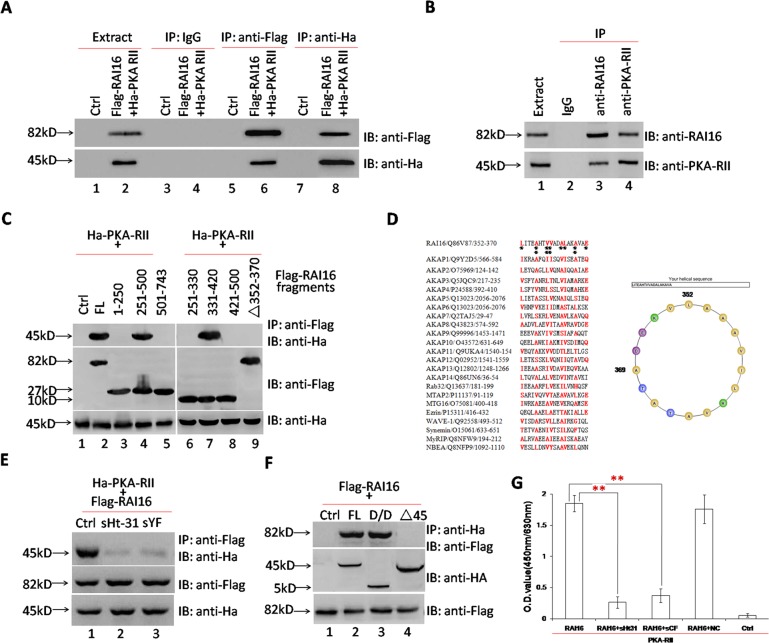
RAI16 interacts directly with PKA-RIIα **A**. RAI16 interacted directly with PKA-RIIα. Flag-tagged RAI16 and Ha-tagged PKA-RIIα were co-expressed in HEK293TT cells. Left panel: Flag-tagged RAI16 was immunoprecipitated with anti-Flag antibody, and interacting proteins were verified with anti-Ha antibody; Right panel: Ha-tagged PKA-RIIα was immunoprecipitated with anti-Ha antibody and interacting Flag-tagged RAI16 were verified with anti-Flag antibody. **B**. Endogenous RAI16 in HEK293TT cells was immunoprecipitated with anti-RAI16 antibody and interacting endogenous PKA-RIIα were verified with anti-PKA-RIIα antibody. **C**. Flag-tagged RAI16 or its fragments or Aa352-370 deletion mutant (Δ352-370) was co-expressed with Ha-tagged PKA-RIIα. Cell lysates were used for immunoprecipitation to verify the interaction. **D**. An amphipathic helical wheel plot for amino acid residues Aa352-370 of RAI16 reveals a hydrophobic surface on one side (Orange). **E**. sHt31 and peptide 348-373 (sYF) could inhibit the interaction of RAI16 and PKA-RIIα. Flag-tagged RAI16 and Ha-tagged PKA-RIIα were co-expressed in HEK293TT cells. Cell lysates were used for immunoprecipitation to verify the interaction. **F**. Flag-tagged RAI16 was co-expressed with Ha-tagged PKA-RIIα full-length (FL), its D/D domain (Aa1-45), or its D/D domain deletion mutant (Δ1-45) in HEK293TT cells. Cell lysates were used for immunoprecipitation to verify the interaction. IB, immunoblot; IP, immunoprecipitation. **G**. Purified PKA-RIIα protein was coated on the microplate, and then was incubated with purified RAI16 protein alone or combinated with sHt31 or sYF, respectively. The interaction was detected by anti-RAI16 antibody and developed with HRP anti-rabbit IgG and TMB.

The interaction of RAI16 and PKA-RIIα was validated in HEK293T cells when recombinant PKA-RIIα was detected in recombinant RAI16 immunoprecipitated complexes (Figure 1A, lower panel, lane 6) or recombinant RAI16 was detected in recombinant PKA-RIIα immunoprecipitated complexes (Figure 1A, upper panel, lane 8). Accordingly, the endogenous proteins were found to interact when RAI16 or PKA-RIIα was immunoprecipitated from the lysates of HEK293T cells (Figure 1B). Next, mapping studies were used to define the interactive surfaces on both RAI16 and PKA-RIIα. Binding of PKA-RIIα to a family of immobilized Flag-RAI16 fragments detected direct interaction with a central portion of RAI16 (Figure 1C).To identify the minimal PKA-RIIα interaction motif on RAI16, we used a Ha-tagged PKA-RIIα fusion protein as a probe to screen a peptide array of overlapping 25-residue peptides (each displaced by three amino acids), spanning the region between residues 334 and 417 of RAI16 (Figure S2A). Specific bindings of Ha-tagged PKA-RIIα were detected only by three peptides, which located in the site between residues 346 and 376 of RAI16 (Figure S2A, upper panel, line 2-4). Thus, the minimal PKA-RIIα interaction region was from residues 352 to 370 (Figure S2A, lower panel, row 2-4). A helical wheel alignment revealed the presence of an amphipathic helix, a hallmark of AKAPs [17], with one side of the helix consisting primarily of hydrophobic residues and the other side of charged/hydrophilic residues, suggesting that RAI16 may be a novel AKAP (Figure 1D, right panel). Similarity between the RAI16 AKB domain and those of AKAPs proved strong (Figure 1D, left panel). sHt31, an anchoring inhibitor peptide, nearly totally disrupted the interaction of RAI16 and PKA-RIIα (Figure 1E, upper panel, lane 2), similar effect was observed by using peptide spYF (peptide of Aa352-370) (Figure 1E, upper panel, lane 3). The D/D domain (Aa1-45) of PKA-RIIα is involved in the interactions of typical AKAPs [18]. Reciprocal experiments demonstrated that RAI16 interacts with the D/D domain of PKA-RIIα (Figure 1F, upper panel, lane 3). The direct interaction of RAI16 and PKA-RIIα, together with the inhibitory effects of sHt31 and sYF, was further confirmed by ELISA assay (Figure 1G). The above findings supported that RAI16 is a novel AKAP.

### RAI16 mediates PKA phosphorylation of HSP70

**Figure 2:**
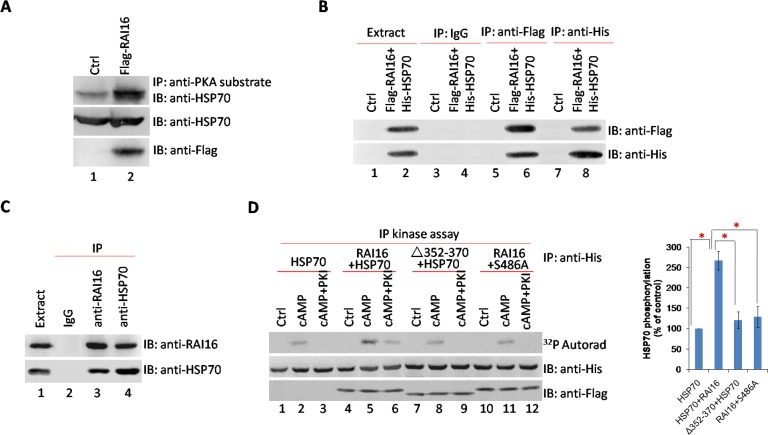
RAI16 mediates PKA phosphorylation of HSP70 **A**. Cell lysates from HEK293T cells expressing Flag-tagged RAI16 or not were immunoprecipitated with anti-PKA substrate antibodies, and endogenous HSP70 were detected. **B**. Flag-tagged RAI16 and His-tagged HSP70 were co-expressed in HEK293TT cells. Cell lysates were used for immunoprecipitation with anti-Flag antibody, anti-His antibody and IgG or not, and interacting proteins were verified with anti-His antibody or anti-Flag antibody. **C**. Endogenous RAI16 in HEK293TT cells was immunoprecipitated with anti-RAI16 antibody and interacting endogenous HSP70 were verified with anti-HSP70 antibody. **D**. HEK293TT cells were co-transfected with His-HSP70 and Flag-RAI16 (lanes 4-6), RAI16 mutant Δ352-370 (lanes 7-9), HSP70 mutant S486A (lanes 10-12) or His-HSP70 alone. Immunoprecipitates with anti-His antibodies were phosphorylated as described in Methods in the absence or presence of 10 mM cAMP (cAMP) or 10 mM cAMP +20 mM PKI (cAMP+PKI_my_r). Proteins were subjected to autoradiography (Left panel). Right panel: Densitometry of the bands corresponding to the phosphorylated His-HSP70 protein that were treated as indicated in left panel. The extent of phosphorylation was normalized to the amount of His-HSP70 present in the immunoprecipitates. Results are the mean of three independent experiments.

HSP70 was initiated for RAI16-binding partners that may also be PKA substrates. We hypothesized that RAI16 targets PKA to phosphorylate HSP70. Lysates from HEK293T cells expressing Flag-tagged RAI16 were incubated with anti-PKA substrate antibodies, immunoprecipitated complexes were isolated and separated by SDS-PAGE. Robust phosphorylation of HSP70 was detected in the presence of RAI16 (Figure 2A, lane 2). The interaction of RAI16 and HSP70 was validated in HEK293T cells when recombinant HSP70 was detected in recombinant RAI16 immunoprecipitated complexes (Figure 2B, lower panel, lane 6) or recombinant RAI16 was detected in recombinant HSP70 immunoprecipitated complexes (Figure 2B, upper panel, lane 8). Accordingly, the endogenous proteins were found to interact when RAI16 or HSP70 was immunoprecipitated from the lysates of HEK293T cells (Figure 2C). Next, mapping studies were used to define the interactive surfaces on both RAI16 and HSP70. Binding of HSP70 to a family of immobilized Flag-RAI16 fragments detected direct interaction with a central portion of RAI16 (Figure S2B, lane 3). Reciprocal experiments demonstrated that RAI16 interacts with C-terminus portion of HSP70 (Figure S2B, lane 8).

HSP70 contains several phosphorylation sites for basophilic kinases, including a well-conserved consensus PKA site surrounding Ser 486 (Table S1B). Treatment of HSP70 immunoprecipitates with cAMP increased by 2.62 fold the phosphorylation of the anchoring protein (Figure 2D, upper panel, lane 5). This effect was totally abolished by PKI, suggesting that it is entirely mediated by PKA (Figure 2D, upper panel, lane 6). The cAMP-induced phosphorylation was abolished in the anchoring-deficient mutant ΔAa352–370 of RAI16 (Figure 2D, upper panel, lane 8), whereas it was strongly reduced in the mutant S486A of HSP70 (Figure 2D, upper panel, lane 11). These results suggest that serine 486 can be phosphorylated by the PKA holoenzyme anchored to HSP70. The increase in phosphorylation of the S486A mutant of HSP70 in response to cAMP, which is blocked by PKI, suggests the existence of a second PKA phosphorylation site, which is less phosphorylated as compared to serine 486.

**Figure 3:**
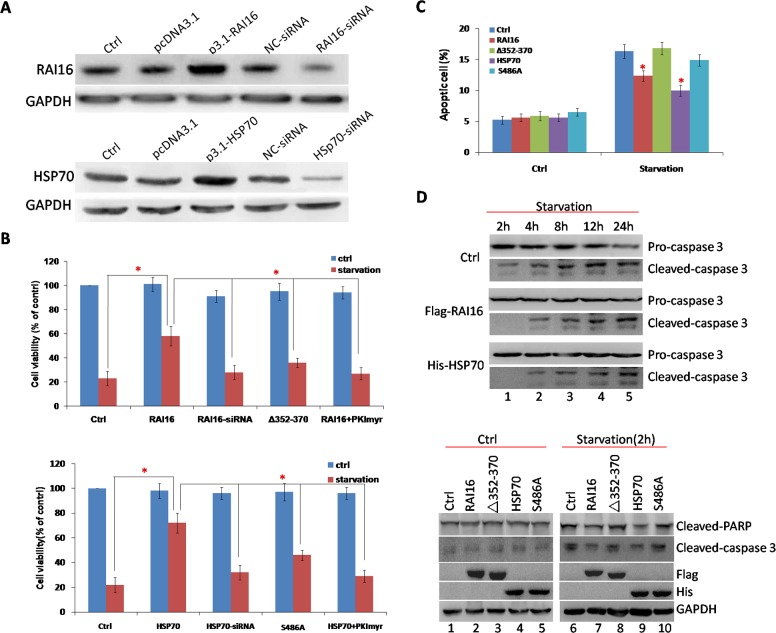
RAI16 was involved in anti-apoptotic effect of HSP70 **A**. RAI16 and HSP70 overexpression or knockdown in HEK293TT cells. **B**. HEK293TT cells expressing RAI16 or HSP70 were tested for proliferation every 24 hours using Cell Titer-Blue cell viability assay (Promega Corp.) according to the manufacturer's instructions. **C**. HEK293TT cells expressing RAI16 or HSP70 were incubated with FITC-Annexin V (Promega Corp.) for 15 minutes at 4°C in the dark and measured using a Cell Lab Quanta SC flow cytometer (Beckman Coulter, Fullerton, CA). **D**. Serum starvation induced cleavage of caspase-3 was inhibited by RAI16 or HSP70 detected by immunoblotting.

HSP70 are known to have special anti-apoptotic properties [19], we investigated the impact of the S486A mutation in HSP70 on anti-apoptosis. Under serum starvation conditions, RAI16 or HSP70 overexpressing cells showed higher cell viability (Figure 3B) and lower cell apoptotic percentage than those of mock cells (Figure 3A). Furthermore, serum starvation induced caspase-3 cleavage was clearly inhibited (Figure 3D, upper panel). The anchoring-deficient mutant ΔAa352–370 of RAI16 and S486A mutant of HSP70 resulted in reduced inhibition (Figure 3D, lower panel).

### 14-3-3θ interacts with RAI16 through a phosphor-serine containing motif

**Figure 4:**
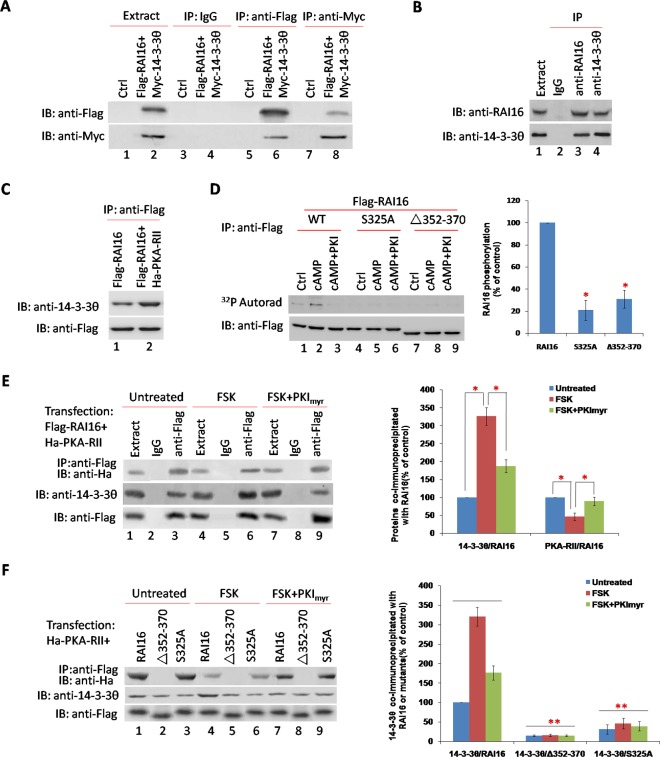
14-3-3θ interacts with RAI16 through a phosphor-serine containing motif **A**. Flag-tagged RAI16 and Myc-tagged 14-3-3θ were co-expressed in HEK293TT cells. Cell lysates were used for immunoprecipitation with anti-Flag antibody, anti-Myc antibody and IgG or not, and interacting proteins were verified with anti-Myc antibody or anti-Flag antibody. **B**. Endogenous RAI16 in HEK293TT cells was immunoprecipitated with anti-RAI16 antibody and interacting endogenous 14-3-3θ were verified with anti-14-3-3θ antibody. **C**. Flag-tagged RAI16 alone or combinated with Ha-tagged PKA-RIIα were expressed in HEK293TT cells. Cell lysates were used for immunoprecipitation with anti-Flag antibody, and interacting endogenous 14-3-3θ were verified with anti-14-3-3θ antibody. **D**. HEK293TT cells were transfected with Flag-RAI16 (lanes 1-3), its mutant S325A (lanes 4-6), or its mutant Δ352-370 (lanes 7-9). Immunoprecipitates with anti-Flag antibodies were phosphorylated as described in Figure 2E. **E**. HEK293TT cells expressing the Flag-tagged RAI16 and Ha-tagged PKA-RIIα were treated for 10 min in the absence (lane 1-3) or presence of 30 mM forskolin (FSK, lane 4-6) or 30 mM FSK+20 mM of myristoylated PKI peptide (FSK+PKI_myr_, lane 7-9). Cell lysates were subjected to immunoprecipitation with anti-Flag antibodie (Left panel). Right panel: Densitometry of the bands corresponding to PKA-RIIα and 14-3-3θ co-immunoprecipitated with RAI16. **F**. HEK293TT cells expressing the Flag-tagged RAI16 (lanes 1, 4, 7) or its mutants Δ352-370 (lanes 2, 5, 8) and S325A (lanes 3, 6, 9) were treated as described previously. Cell lysates were subjected to immunoprecipitation with anti-Flag antibodie (Left panel). Right panel: Densitometry of the bands corresponding to 14-3-3θ co-immunoprecipitated with RAI16 or its mutants.

14-3-3θ was also detected in RAI16 immunoprecipitated complexes. The interaction of RAI16 and 14-3-3θ was validated in HEK293T cells when recombinant 14-3-3θ was detected in recombinant RAI16 immunoprecipitated complexes (Figure 4A, lower panel, lane 6) or recombinant RAI16 was detected in recombinant 14-3-3θ immunoprecipitated complexes (Figure 4A, upper panel, lane 8). Accordingly, the endogenous proteins were found to interact when RAI16 or 14-3-3θ was immunoprecipitated from the lysates of HEK293T cells (Figure 4B). In addition, immunoprecipitated 14-3-3θ in HEK293T cells expressing combination of RAI16 and PKA-RII was significantly higher than that in HEK293T cells expressing only RAI16, which indicated that the interaction of RAI16 and 14-3-3θ was dependent on the RAI16/PKA-RII complex (Figure 4C). Next, mapping studies were used to define the interactive surfaces on both RAI16 and 14-3-3θ. Binding of 14-3-3θ to a family of immobilized Flag-RAI16 fragments detected direct interaction with a central portion of RAI16 (Figure S2C, lane 3). Reciprocal experiments demonstrated that RAI16 interacts with a central portion of 14-3-3θ (Figure S2C, lane 7).

Members of the 14-3-3 family interact with several cellular targets through the association with phosphor-serine or phosphor-threonine containing motifs [20]. Phosphorylation of these motifs is catalyzed predominantly by members of the AGC subfamily of protein kinases such as PKA, PKB and PKC [21]. Since RAI16 is a PKA-binding protein, we hypothesized that PKA might phosphorylate RAI16 and regulate its interaction with 14-3-3θ. Serine 325 within the 14-3-3θ binding motif of RAI16 is predicted to form a phosphorylation site for basophilic kinases (Table S1A). Based on our findings that phosphorylation of RAI16 by PKA is required for the association of 14-3-3θ, we investigated whether PKA might directly phosphorylate serine 325. Treatment of RAI16 immunoprecipitates with cAMP increased by 2.87 fold the phosphorylation of the anchoring protein (Figure 4D, upper panel, lane 2). The cAMP-induced phosphorylation was completely abolished by S325A mutation or Aa352-370 deletion (Figure 4D, upper panel, lane 5 and 8). These results suggest that serine 486 can be phosphorylated by the PKA holoenzyme anchored to HSP70. No phosphorylation was observed in the presence of the PKA specific inhibitor PKI (Figure 4D, upper panel, lane 3, 6, 9).

To demonstrate that the phosphorylation of serine 325 of RAI16 by anchored PKA is required for the association with 14-3-3θ. Treatment of forskolin (FSK) enhanced the association of 14-3-3θ to Flag-RAI16 by 3.25–fold (Figure 4E, medium panel, lane 6), whereas pretreatment with PKI_myr_ (FSK+PKI_myr_) strongly inhibited this interaction (Figure 4E, medium panel, lane 9). Accordingly, both the ΔAa352-370 mutant of RAI16 were impaired in its ability to associate with 14-3-3θ either under basal conditions or after FSK treatment (Figure 4F, middle panel, lanes 2,5,8), whereas S325A mutant abolished the interaction of RAI16 with 14-3-3θ (Figure 4F, middle panel, lanes 3,6,9). These results strongly suggest that phosphorylation of serine 325 by the PKA holoenzyme associated with RAI16 is required for the binding of 14-3-3θ to the anchoring protein. Interesting, Treatment of FSK reduced the association of RAI16 to PKA RII by 0.45-fold (Figure 4E, upper panel, lane 6 and Figure 4E, upper panel, lane 4), whereas pretreatment with PKImyr (FSK+PKI_myr_) strongly inhibited this interaction (Figure 4E, upper panel, lane 9 and Figure 4F, upper panel, lane 7).

### 14-3-3θ inhibits RAI16 medicated PKA phosphorylation of HSP70

**Figure 5:**
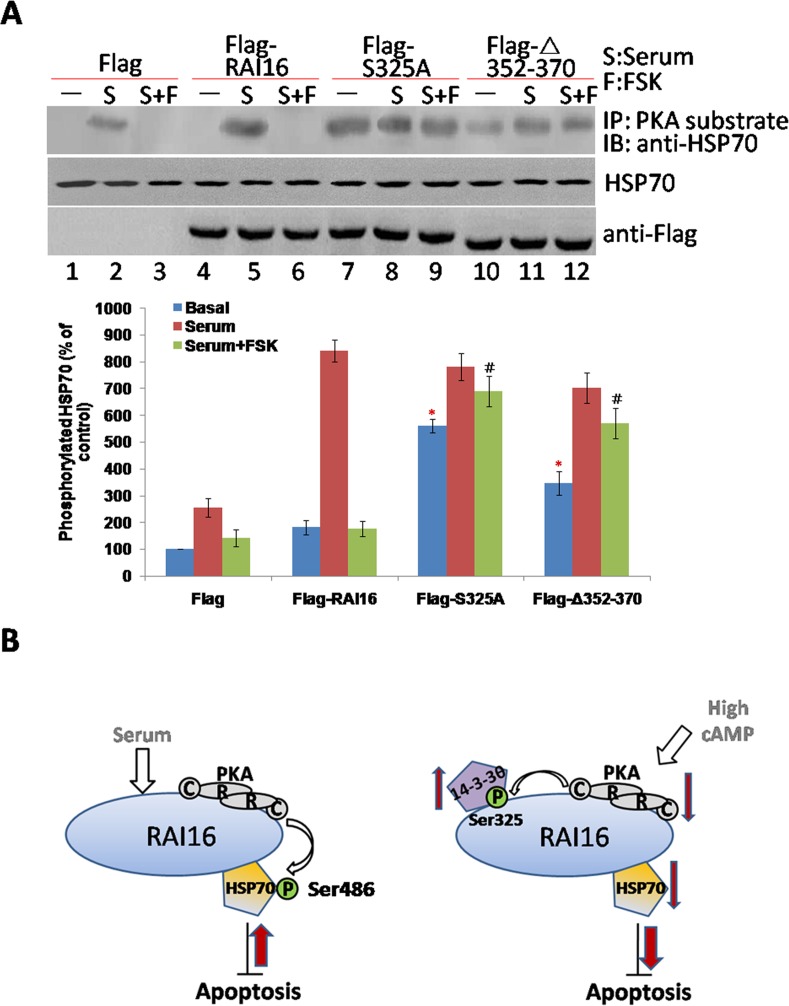
14-3-3θ inhibits RAI16 medicated PKA phosphorylation of HSP70 **A**. HEK293TT cells expressing Flag (lanes 1–3) or the Flag-tagged RAI16 (lanes 4–6) or of its mutants S32A (lanes 7–9) and Δ352-370 (lanes 10–12) were serum starved for 12 h, and then treated for 1 h in the absence or presence of 10% fetal calf serum (S) or 10% fetal calf serum+10 mM forskolin (S+FSK). Cell lysates were immunoprecipitated with anti-PKA substrate antibodies, and endogenous HSP70 were detected (Upper panel). Lower panel: Densitometry of the bands corresponding to HSP70 co-immunoprecipitated with RAI16 (normalized to the HSP70 of cell extracts). **B**. Model for the 14-3-3θ mediated inhibition of RAI16. Left panel: RAI16 is activated in response to serum stimulation. RAI16 anchored PKA to phosphorylate HSP70 on serine 408, which enhances the anti-apoptosis activity of HSP70. Right panel: Elevation of the cellular concentration of cAMP activates the PKA holoenzyme anchored to RAI16. The catalytic subunit released from the PKA holoenzyme can phosphorylate RAI16 on serine 325. This induces the recruitment of 14-3-3θ, which inhibits RAI16 mediated PKA phosphorylation of HSP70, then the anti-apoptosis activity of HSP70.

To determine whether 14-3-3θ can inhibit RAI16 medicated phosphorylation of HSP70. RAI16 displays a low basal expression in serum-starved cells and can be upregulated by treatment of serum (data not shown). Accordingly, treatment of 10% serum strongly stimulated RAI16 phosphorylation of HSP70 (Figure 5A, upper panel, lane 5), whereas FSK treatment inhibited by 75% this stimulatory effect (Figure 5A, upper panel, lane 6), suggesting that stimulation of the PKA pathway can inhibit the activation of RAI16 induced by serum. Remarkably, both the S325A and ΔAa352–370 mutants of RAI16 displayed basal phosphorylation of HSP70 significantly higher than wild-type RAI16 (Figure 5A, upper panel, lanes 7,10) and showed an increased resistance to FSK-mediated inhibition (Figure 5A, upper panel, lanes 9,12). These results suggest that anchoring of PKA and binding of 14-3-3θ to RAI16 are both required to maintain the anchoring protein in a low activity state under basal unstimulated conditions.

The model presented in Figure 5B depicts that RAI16 mediates PKA phosphorylation of HSP70, and RAI16 also could be phosphorylated by the anchored PKA, that promotes the recruitment of 14-3-3θ, which, in turn, inhibits RAI16 mediated PKA phosphorylation of HSP70. Although we have not yet established the stoichiometry of the components in this network, our findings provide evidences that RAI16 phosphorylation of HSP70 is regulated by the anchoring of both PKA and 14-3-3θ to RAI16.

## DISCUSSION

There are four genetically distinct and functionally isoforms of PKA regulatory subunits: RIα, RIβ, RIIα, and RIIβ in mammalian cells, which all contain a D/D domain capable of AKAP binding. Most reported AKAPs have a preferred specificity for PKA-RII, whereas three AKAPs: D-AKAP1 [22], D-AKAP2 [23] and Opa1 [24] are dual-specific AKAPs, and SPHKAP is the PKA-RI-specific AKAP [25, 26]. Here we identified that RAI16 is a typical RII specific AKAP (RAI16 couldn't interact with RIα, RIβ, or RIIβ detected by Co-IP, data not shown). The Aa352–370 fragment of RAI16, forming an amphipathic helix, specifically docks to the D/D domain of PKA-RIIα. Deletion of either the RAI16 Aa352–370 region or the RIIα D/D domain completely abolished the interaction. The customary function of AKAP is to compartmentalize PKA holoenzyme to the preferred substrates [5]. Our results confirm this concept that RAI16 functions as an adaptor protein, medicating PKA phosphorylation of HSP70 at Ser486.

It is well known, as a molecular chaperone, Hsp70 is an important part of cellular networks, including transcriptional, signaling, membrane and organelle networks [28]. In addition, constitutive high levels of Hsp70 are frequently observed in various cancer cells [29, 30], and correlated with increased cancer cell proliferation [31], clinical stage [32, 33] or increased grade and shorter overall survival [34]. Hsp70 enhances cell growth, suppresses senescence, and confers resistance to stress-induced apoptosis and serves as a good tumor marker to identify patients with early-stage prostate cancer and hepatocellular carcinoma [35, 36]. Here, we provided new evidence that the phosphorylation of HSP70 at Ser486 was important for anti-apoptosis induced by serum starvation. Similar findings on PKA phosphorylation of HSP20 by AKAP-Lbc has been reported to be involved in cardioprotective effects [27], thus, phosphorylation of HSP family by PKA /AKAP might play important roles in diverse cell functions. Moreover, HSP70 has been reported to be phosphorylated by PKC [37, 38]. Although, we failed to identify PKC as the interacting partner of RAI16 by mass spectrometry analysis in this study, whether RAI16 interacts with PKC and is involved in phosphorylation of HSP70 by PKC needs further study.

Additionally, we show that RAI16 itself was phosphorylated at Ser325 by PKA, which is also the binding site for 14-3-3 protein. 14-3-3 proteins are highly conserved from yeast to human and consist of seven mammalian isoforms (b, e, g, c, t/h, f and s) [39]. 14-3-3 proteins are known to interact with over 200 proteins with specific phosphor-serine/threonine motifs [40]. Through binding to their target proteins, 14-3-3 participates in a wide variety of biological processes [41-43]. The present study found that 14-3-3θ interacted with phosphorylated RAI16, resulting in inhibition of RAI16 mediated PKA phosphorylation of HSP70. This finding seems controversial to previous reported anti-apoptosis effect of 14-3-3 [44-46], suggesting more complicated mechanisms of 14-3-3 regulation.

Conclusively, the implication of our study is that we firstly demonstrate the functional role of RAI16 as an AKAP. Our findings suggested RAI16 might play a role in HSP70 related anti-apoptosis pathway. We also demonstrate that binding of 14-3-3θ to RAI16 maintains the anchoring protein in an inactive state under basal conditions.

## MATERIALS AND METHODS

### Cell culture, transfection, cell viability, cell apoptosis assays and immunoblotting

Human embryonic kidney cell line HEK293T was cultured and performed transfection, cell viability, cell apoptosis assays and immunoblotting as described previously [16].

### Plasmids construction

The full length gene of PKA-RIIα (GeneBank: NM_004157.2), HSP70 (GeneBank: NM_005345.5) and 14-3-3θ (GeneBank: NM_006826.3) were PCR amplified from total RNA of HEK293T cell as described previously [16] and cloned into pMD18T vector (Takara), termed as p18T-PKA-RIIα, p18T-HSP70 and p18T-14-3-3θ, respectively.

Fragments of 1-45 and full length gene of PKA-RIIα were PCR amplified from the p18T- PKA-RIIα plasmid and subcloned into pcDNA3.1 with a Ha tag. Fragments of 1-250, 251-500 and 501-743 and full length gene of RAI16 were PCR amplified from the p18T-RAI16 plasmid [16] and subcloned into pcDNA3.1 with a flag tag. Fragments of 1-220, 221-440 and 441-641 and full length gene of HSP70 were PCR amplified from the p18T-HSP70 plasmid and subcloned into pcDNA3.1 with a His tag. Fragments of 1-80, 81-160 and 161-245 and full length gene of 14-3-3θ were PCR amplified from the p18T-14-3-3θ plasmid and subcloned into pcDNA3.1 with a Myc tag.

The S325A and Δ352-370 mutants of RAI16, S486A mutant of HSP70, Δ1-45 mutant of PKA-RIIα were introduced into the corresponding plasmids by standard PCR directed mutagenesis using the Hot Star DNA polymerase (Qiagen), respectively.

### Immunoprecipitation, mass spectrometry analysis and phosphorylation assay

Immunoprecipitation and *in vitro* phosphorylation assay was performed as reported previously [47]. All proteins were subjected to trypsin digestion and peptides were identified by MS/MS sequencing on LTQ Orbitrap instrument as described previously [48].

### Expression recombinant proteins in E.coli and Protein-protein interactions by ELISA

His-tagged fusion proteins of RAI16 and PKA-RIIα were expressed using pET30 in BL21DE3 bacteria and purified with Nickel-NTA chelating resin (Amersham Pharmacia Biotech) according to manufactures' protocol. The direct interaction of RAI16 and PKA-RIIα were studied by ELISA. In brief, 2ug purified PKA-RIIα protein was coated overnight at 4°C. Following a blocking with 3% BSA in PBST, purified RAI16 protein alone or combinated with stH31 or sYF were added to microplate and incubated for 1h at 37°C, and then washing 5 times with PBST, anti-RAI16 antibody was added to detect the interaction, HRP-anti-rabbit IgG and TMB (HRP substrate) were used for signal development. The values were recorded using a multiplate reader (Synergy 2, BioTek).

### Antibodies and reagents

RAI16 and PKA-RIIα antibodies were from Abcam; 14-3-3θ, Caspase-3, Flag and Myc epitope antibodies were from ABclone; HA and His epitope antibodies were from MBL; HSP70 and GAPDH antibodies were from Santa Cruz Biotechnology; Anti-PKA substrate antibody were from Cell Signaling. RAI16-siRNA, HSP70-siRNA and nonrelative control (NC) siRNA were synthesized by GenePharma (Shanghai, China); FSK were purchased from Sigma; PKI_myr_, sHt31 were from Promega. Peptide sYF (Aa348-373: YCDHLITEAHTVVADALAKAVAENF) was synthesized by ABclone (Shanghai, China).

### Motif prediction

14-3-3 binding site in RAI16 and phosphorylation sites in RAI16 and HSP70 were predicted by Scansite Motif Scanner (http://scansite.mit.edu/motifscan_seq.phtml).

### Statistical analysis

All experiments were performed at least three times, and data are presented as mean ± SD. Comparisons were made by using a two-tailed t test or one-way ANOVA for experiments with more than two subgroups. P<0.05 was considered to be statistically significant.

## SUPPLEMENTARY MATERIAL, FIGURES AND TABLE


