# Supplementary material for: Anchoring of both PKA-RIIα and 14-3-3θ regulates retinoic acid induced 16 mediated phosphorylation of heat shock protein 70
Source: Oncotarget. 2015 Mar 30;6(17):15540–50. doi: 10.18632/oncotarget.3702 (PMC4558169; doi:10.18632/oncotarget.3702)
Supplement: Supplementary file 1 [file oncotarget-06-15540-s001.pdf]

# Anchoring of both PKA-RIIα and 14-3-3θ regulates retinoic acid induced 16 mediated phosphorylation of heat shock protein 70

**Supplementary Material**

**A. Interacting proteins of RAI16 identified by MS/MS.**

| Protein Mass | No. of Peptide | gene symbols | Link | Relative Abundance |
|---|---|---|---|---|
| 83200.58 | 12 | FAM160B2 | Q86V87 | 2.8% |
| 78017.97 | 10 | HSP70A1 | Q59EJ3 | 1.7% |
| 27764.32 | 6 | 14-3-3θ | P27348 | 1.6% |
| 43066.75 | 3 | PKA-RIIα | P13861 | 0.2% |

**B. Representative peptides of RAI16 and HSP70 identified by MS/MS.**

| Peptide Mass | Peptide Sequence | Sequence Header |
|---|---|---|
| 1042.51 | ACGEPTALPK | >sp\|Q86V87\|F16B2_HUMAN Protein FAM160B2 |

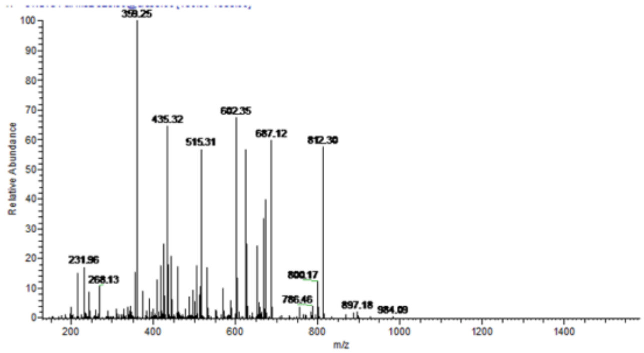


| Peptide Mass | Peptide Sequence | Sequence Header |
|---|---|---|
| 1486.69 | TIPSYVAFTDTER | >tr\|Q59EJ3\|Q59EJ3_HUMAN Heat shock 70kDa protein 1A variant (Fragment) O |

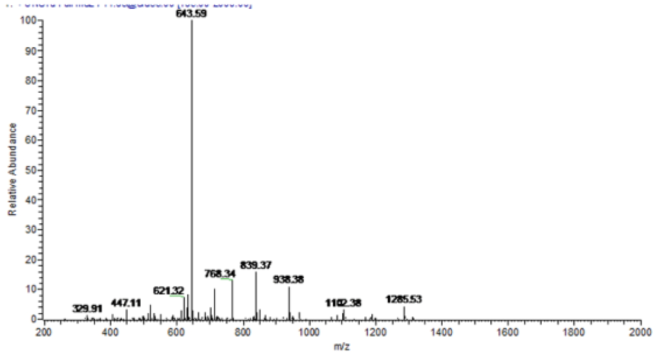


**Figure S1**

**Figure S1.**

A. Interacting proteins of RAI16 identified by MS/MS in this study.

B. Representative peptides of RAI16 and HSP70 identified by MS/MS.

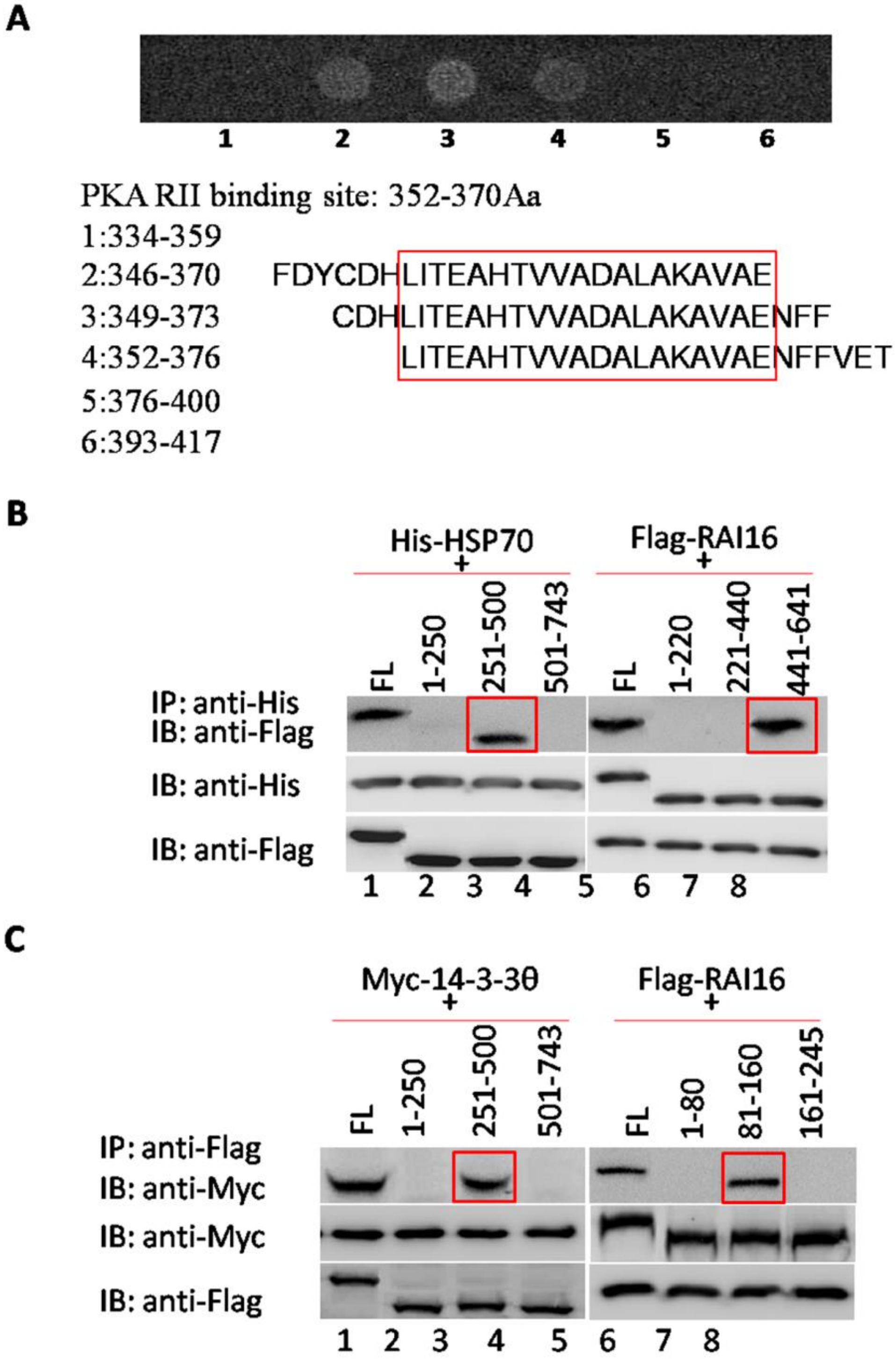


**Figure S2**

**Figure S2.**

**A**. The representatives of peptide array analysis of the PKA-RIIα binding site on RAI16. The results of three peptides (row 2-4) located around Aa352-370 region, one peptide (row 1) located in front of Aa352-370 region and two peptides (row 5-6) located behind Aa352-370 region were showed.

**B**. Flag-tagged RAI16 or its fragments were co-expressed with His-tagged HSP70 or His-tagged HSP70 or its fragments were co-expressed with Flag-tagged RAI16. Cell lysates were used for immunoprecipitation to verify the interaction.

**C**. Flag-tagged RAI16 or its fragments were co-expressed with Myc-tagged 14-3-3θ or Myc-tagged 14-3-3θ or its fragments were co-expressed with Flag-tagged RAI16. Cell lysates were used for immunoprecipitation to verify the interaction.

**Table S1: Motif analysisof RAI16 and HSP70 by Scansite Motif Scanner.**

## A. 14-3-3 binding and phosphorylation sites of RAI16

| Phosphoserine/threonine binding group (pST_bind) | | | | |
|---|---|---|---|---|
| Site | Score | Percentile | Sequence | SA |
| **S325** | 0.3593 | 0.243 % | GISWRLPSAPSDEAS | 0.820 |
| **Basophilic serine/threonine kinase group (Baso_ST_kin)** | | | | |
| Site | Score | Percentile | Sequence | SA |
| S579 | 0.5264 | 0.355 % | ECSSRVASWGWPLTP | 0.252 |
| Site | Score | Percentile | Sequence | SA |
| **S325** | 0.5718 | 0.705 % | GISWRLPSAPSDEAS | 0.820 |
| Site | Score | Percentile | Sequence | SA |
| S128 | 0.4187 | 0.165 % | HPLLHYLSVHRPVQK | 0.785 |

## B. Phosphorylation sites of HSP70

| Basophilic serine/threonine kinase group (Baso_ST_kin) | | | | |
|---|---|---|---|---|
| Site | Score | Percentile | Sequence | SA |
| T487 | 0.4196 | 0.114 % | ALIKRNSTIPTKQTQ | 1.158 |
| Site | Score | Percentile | Sequence | SA |
| **S486** | 0.4352 | 0.605 % | TALIKRNSTIPTKQT | 1.571 |
| Site | Score | Percentile | Sequence | SA |
| S343 | 0.5045 | 0.693 % | ERAKRTLSSSTQASL | 0.947 |

*:http://scansite.mit.edu/motifscan_seq.phtml
